# Interactions between Phase-Separated Liquids and Membrane Surfaces

**DOI:** 10.3390/app11031288

**Published:** 2021-01-31

**Authors:** Samuel Botterbusch, Tobias Baumgart

**Affiliations:** 1 Department of Chemistry, University of Pennsylvania

**Keywords:** liquid-liquid phase separation, biomimetic membranes, aqueous two-phase systems, biomolecular condensates, complex coacervation

## Abstract

Liquid-liquid phase separation has recently emerged as an important fundamental organizational phenomenon in biological settings. Most studies of biological phase separation have focused on droplets that “condense” from solution above a critical concentration, forming so-called “membraneless organelles” suspended in solution. However, membranes are ubiquitous throughout cells, and many biomolecular condensates interact with membrane surfaces. Such membrane-associated phase-separated systems range from clusters of integral or peripheral membrane proteins in the plane of the membrane to free, spherical droplets wetting membrane surfaces to droplets containing small lipid vesicles. In this review, we consider phase-separated liquids that interact with membrane surfaces and we discuss the consequences of those interactions. The physical properties of distinct liquid phases in contact with bilayers can reshape the membrane, and liquid-liquid phase separation can construct membrane-associated protein structures, modulate their function, and organize collections of lipid vesicles dynamically. We summarize the common phenomena that arise in these systems of liquid phases and membranes.

## Introduction

Liquid-liquid phase separation and the formation of biomolecular condensates have recently been identified as important contributors to cellular organization. Phase-separated condensates are implicated in more and more biological structures: signaling complexes[1–3], the nucleolus[4,5], P granules[6] and nuage[7], the nuclear pore complex[8], stress granules[9], and many others[10]. As such, the body of scientific work characterizing this collection of biomolecular condensates is ever-expanding. These entities have been of scientific interest not only because of their seemingly widespread occurrence, but also because of their versatility in function: Biomolecular condensates are responsive to stimuli, concentrate biomolecules with specificity, buffer protein concentrations, and nucleate larger cellular structures, among other functions[11]. Beyond this diversity of function, liquid-liquid phase separation attracts attention because it is a mechanism for the formation of so-called “membraneless organelles[8].” In this way it provides a principle of cellular organization distinct from traditional, membrane-bound organelles[10,12].

Membranes are ubiquitous in cells, and many biomolecular condensates interact with membrane surfaces. These hybrid phase-separated condensate and membrane systems appear in different forms, with different consequences for the function of the biomolecular and membrane components. As we will show in this review, they range from clusters of integral or peripheral membrane proteins in the plane of the membrane to spherical droplets wetting membrane surfaces to droplets containing small lipid vesicles.

In this review, we consider phase-separated liquids that interact with membrane surfaces and we discuss the consequences of those interactions. The functions performed by biomolecular condensates at membrane surfaces are broad. We will first examine how membranes may be reshaped and laterally reorganized through contact with distinct liquid phases with different physical properties. Then we will turn our attention to how liquid-liquid phase separation can have a role in constructing membrane-associated protein structures and controlling their function. Finally, we will discuss the organization of collections of lipid vesicles by biomolecular condensates and how active processes and other cellular regulatory mechanisms modulate the behavior of membrane-associated condensates. Throughout, our focus will be on experiments using biomimetic membranes. However, at times discoveries made *in silico* or in cells or cell-derived membranes will be presented to better contextualize the state of research in this area.

### 1. Physical Properties of Phase-Separating Molecules and Biomolecular Condensates

Liquid-liquid phase separation has been the focus of a large body of scientific work in the last several years, and as such a high volume of information is available. Multiple reviews have covered in detail common properties many phase-separating molecules or the biomolecular condensates formed from them share[10,12–14]. Here, we will provide an overview of those factors influencing phase separation and the physical properties biomolecular condensates possess as a result. Many of these properties inform the basic behavior of biomolecular condensates in a cellular environment, including at the membrane. This summary is by no means comprehensive but should provide a sense of the dynamic behaviors of biomolecular condensates and what kinds of functionality they can provide to the cell.

#### 1.1. Biophysical Drivers of Liquid-Liquid Phase Separation

The basic principles of biomolecules condensing into a distinct liquid phase are well modeled by polymer physics. The Flory-Huggins model calculates the free energy of a solution of homopolymers and assumes a simple parameter χ—the Flory-Huggins parameter—dependent on the strength of solvent-solvent, solvent-polymer, and polymer-polymer interactions that has been used to predict polymer phase separation at high χ[14–16]. Overbeek and Voorn provide a model that predicts phase separation between polyions dependent on polyion length and linear charge density[14,17]. Both of these models, however, rely on polymers of homogeneous composition. More realistic models of biomolecule phase separation explore the effects of sequence heterogeneity and specific interaction domains.

A wide variety of structural factors may promote or inhibit the ability of a protein to phase separate. For example, many phase-separating proteins contain intrinsically disordered regions (IDRs) that often drive phase separation. The proteins Ddx4 and LAF-1, both associated with P-granules—membraneless organelles implicated in germ cell lineage maintenance[6,7,12]—contain such IDRs. Archetypes of IDRs that phase separate include IDRs like that of FUS[18] enriched in polar residues, those like Ddx4[7] containing oppositely-charged “blocks” of charged residues, and those like NICD that carry a net charge that drives condensation with another, oppositely-charged, molecule[12,19]. Folded, interactive domains can also lead to the formation of biomolecular condensates. In these systems, multivalency—that is, repeats of specific binding motifs or domains in a single molecule—is known to be an important factor promoting phase separation[2,12]. The linkers between these domains also affect a protein’s ability to phase separate: Weak interactions involving disordered linkers can be a necessary contributor to phase separation[20] and the length and solvent affinity of linkers can impact whether a protein experiences phase separation with increasing concentration before undergoing gelation[21]. The ways in which domains and linkers impact the physics of biomolecular condensate formation and their physical properties have been investigated thoroughly through a “stickers and spacers” model which builds on Semenov and Rubenstein’s mean-field theory for associative polymers[22]. Choi et al. have recently reviewed this model[13].

#### 1.2. Stimulus Responsiveness of Biomolecular Condensates

**Figure 1.**
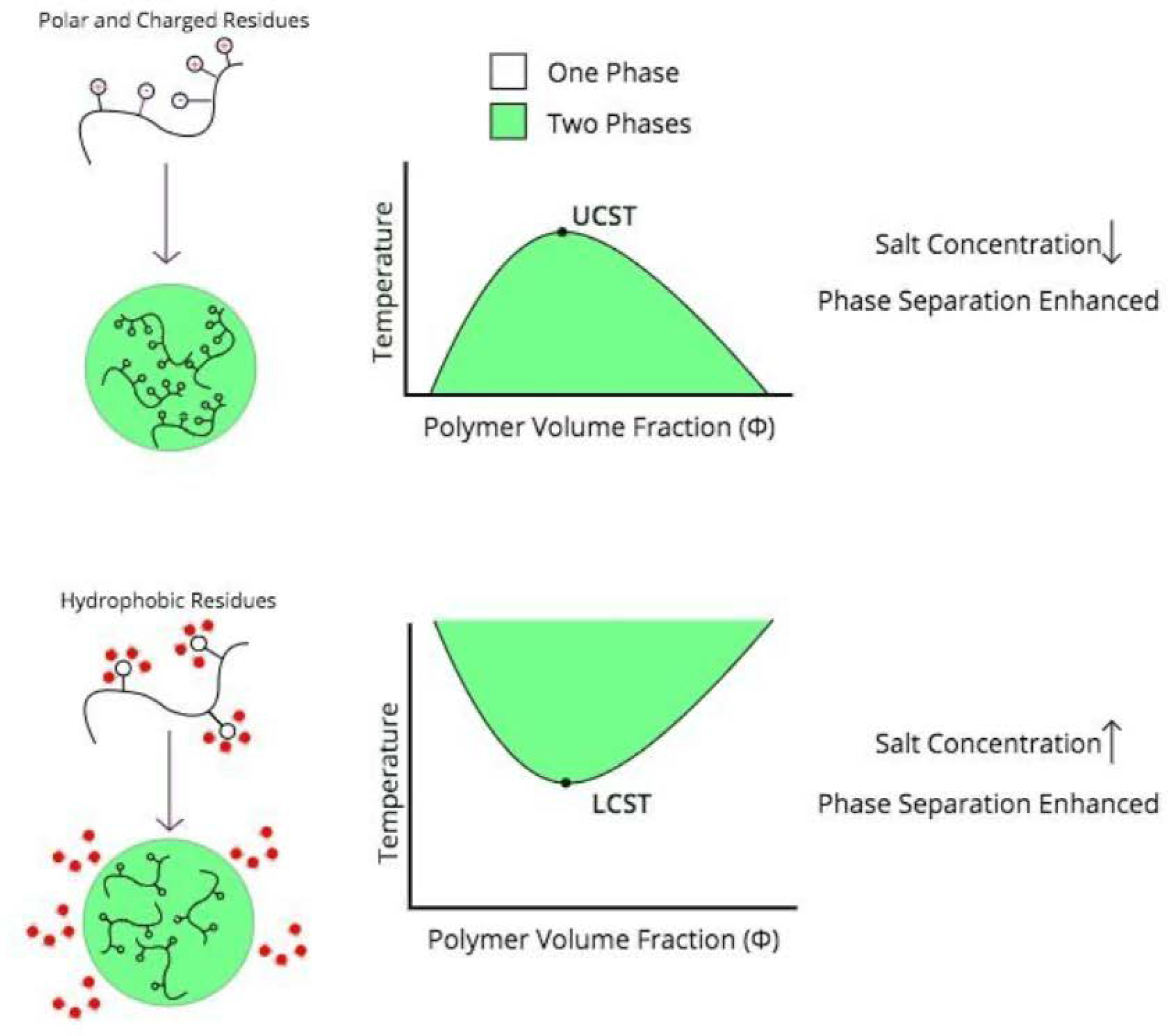
Trends in stimulus responsiveness of phase-separated condensates. There are exceptions depending on the precise thermodynamics of the polymer phase separation; for example, FUS LC phase separates at low temperatures but at high salt concentrations[18].

Because liquid-liquid phase separation occurs in a precise thermodynamic regime, biomolecular condensate formation and dissolution is intrinsically responsive to stimuli that shift the system in and out of these regimes. However, how biomolecular condensates respond to stimuli depends on the nature of the intermolecular interactions that generate them (Fig. 1). Temperature, naturally, affects the thermodynamics of phase separation. In Flory-Huggins theory, phase separation is enthalpically driven, and so theoretically simple polymer condensates have an upper critical solution temperature above which phase separation will not occur[23]. However, many phase-separating systems instead have a lower critical solution temperature below which phase separation does not occur. These polymer condensates are often entropically driven by the release of water from hydrophobic polymer surfaces[24]. Hydrophobic residue-rich proteins such as elastin-like polypeptides and the protein BuGZ phase separate with increased temperature, and indeed BuGZ mutants replacing aromatic residues with the polar residue serine require much higher temperature and concentration to phase separate[24,25]. Ddx4 condensates on the other hand, which are driven by electrostatic interactions, form at lower temperatures and are dissolve upon heating[7].

Like temperature, ionic strength can either enhance or inhibit phase separation depending on the nature of intermolecular interactions. LAF-1, like Ddx4, undergoes phase separation driven by electrostatic interactions, and both experience an inhibitory effect from salt—increased salt concentrations increase the critical protein concentration for phase separation[6,7]. Proteins such as tropoelastin and FUS, which form biomolecular condensates due to hydrophobic interactions, can “salt out” of solution in a way that promotes liquid-liquid phase separation at elevated salt concentrations[18,26,27]. It should be noted that though FUS’s low-complexity domain (FUS LC) undergoes hydrophobic residue-mediated phase separation that is enhanced by ionic strength, it is lower temperatures rather than higher that promote its phase separation[18]. These principles of stimulus responsiveness based on the type of intermolecular interactions are only guidelines and the specific thermodynamics of phase separation vary from case to case.

### 2. Contact with Liquid Phases that Separate from Solution Reshapes Membranes at Phase Boundaries

Aqueous two-phase systems (ATPSs) encapsulated by a liposome represent a useful model for understanding the basic physical interactions between a lipid membrane and a liquid-liquid phase boundary. An aqueous solution of dextran and polyethylene glycol (PEG) can separate into an ATPS under appropriate experimental conditions, and this transition can be reversibly induced within vesicles by changing temperature[28,29] and osmolarity[29]. These two liquid phases reshape and are reshaped by the encapsulating membrane due to the interfacial forces between the liquid bulk phases and the membrane.

#### 2.1. Wetting Transition & Budding in Vesicle-Enclosed ATPSs

**Figure 2.**
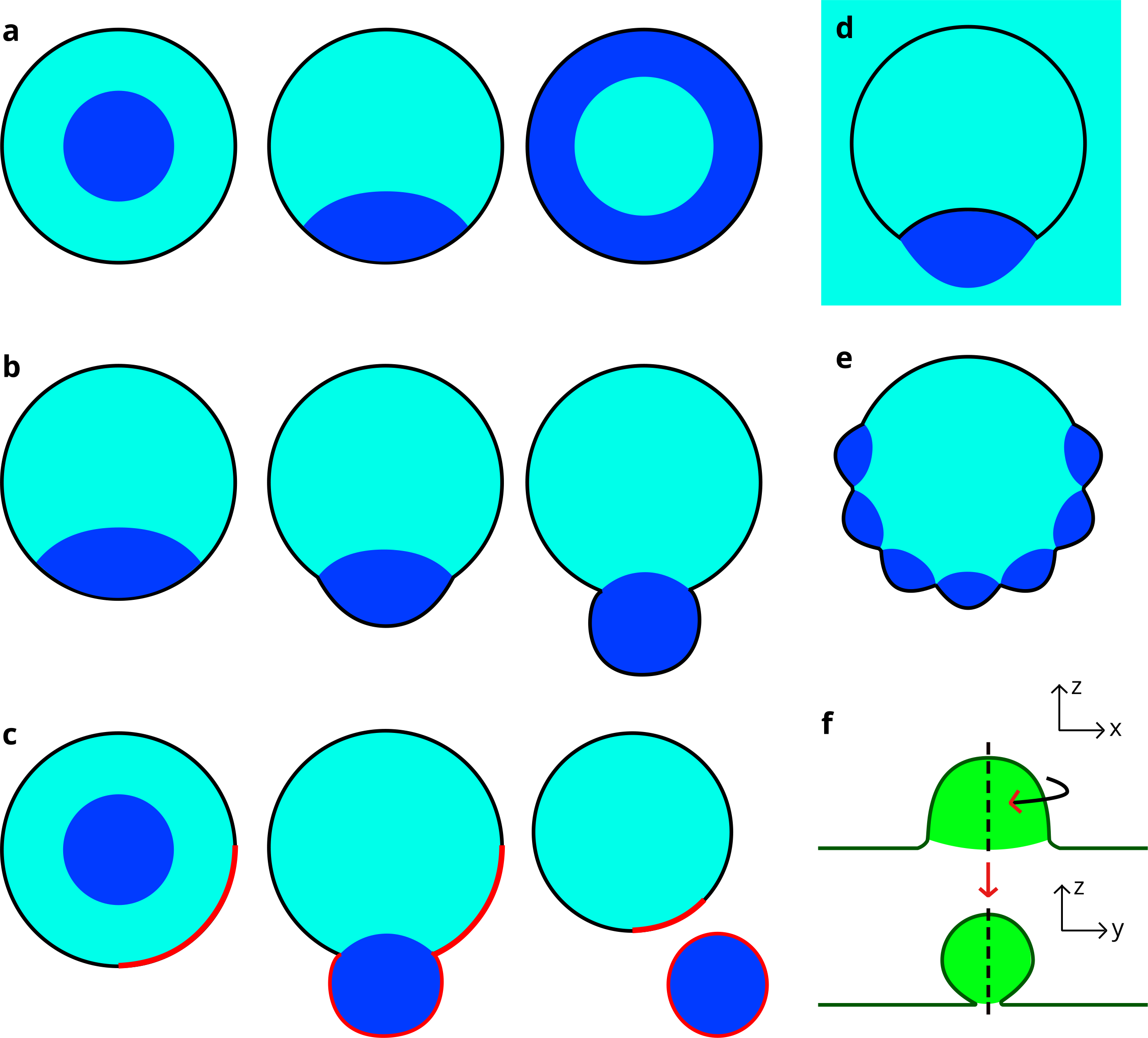
**(a)** Wetting transition leading to inversion of liquid phases; **(b)** Wetting-induced budding, potentially leading to **(c)** complete vesicle fission. Note that in the presence of a heterogeneous membrane, the “red” lipid phase has an affinity for the dark blue liquid phase, and the two bud and separate from the mother vesicle together; **(d)** “Inward” budding induced by a dark blue liquid droplet external to the vesicle; **(e)** Raspberry-like vesicle morphology caused by several smaller droplets wetting the membrane; these droplets will coalesce to form a single bud; **(f)** During membrane engulfment of a nanodroplet, symmetry is broken; the line tension is negative so a long contact line minimizes line tension energy while the contact area between the separate liquid phases is reduced, creating a “lipped” membrane neck. The plane of the upper cross section is shown by the dotted line below and vice versa.

Wetting of a membrane by a liquid phase—constituting a modification of the contact area between the phase and the membrane as the liquid coats the surface—is dependent on properties of the ATPS. In a system with a fixed PEG to dextran weight ratio, raising the total polymer concentration increased the membrane wetting by the dextran phase, from no wetting (full PEG wetting) to partial wetting[30]. Similarly, vesicles that formed with PEG bordering the lipid membrane and dextran as a droplet in the center saw a reversal upon hypertonic dehydration: The PEG phase moved to the center as dextran fully bordered the membrane (Fig. 2a)[29].

Other experiments using osmotic dehydration to generate an ATPS displayed a deflation effect that reduced the vesicle volume. This generated excess membrane area relative to the enclosed volume, allowing the vesicle to take on non-spherical shapes and leading to liquid phase droplets and membrane reshaping one another[31]. Many studies identified budding—a nonspherical morphology in which a “bud” deviates from the spherical membrane and partially surrounds one of the liquid phases (Fig. 2b)—in these systems as a reshaping phenomenon[28,31–34]. Budding occurred while the dextran droplet wetted the vesicle interior, as the liquid-liquid interfacial tension pulled inward on the membrane[34]. In some cases this reshaping led to complete division into two smaller vesicles. If distinct lipid domains were present in the membrane prior to division, these would partition into daughter vesicles containing different bulk liquid phases (Fig. 2c)[33]. In addition to these reshaping processes, “inward” budding, where external dextran droplets wet and bend inward the vesicle membrane, has been observed (Fig. 2d)[34]. Other, more irregular membrane shapes such as “raspberries” of small droplet buds have formed during deflation (Fig. 2e), though those smaller droplets eventually coalesced into a larger one forming a single bud in a vesicle[31].

#### 2.2. Deviations from Budding: Nanotubes and Nanodroplets

Budding is in competition with other reshaping phenomena, depending on the spontaneous curvature of the vesicle membrane[34]. Internal membrane nanotubes have been observed in deflated vesicles that contained a PEG/dextran ATPS; the appearance of these tubules implies spontaneous curvature must have been induced[31]. These tubes preferentially located to the liquid-liquid phase boundary[31,35]. Spontaneous curvature in nanotube-forming vesicles is a result of membrane-polymer interactions and the magnitude of this induced curvature depends on the phase of the vesicle membrane: Liquid-disordered membranes obtained far more curvature than liquid-ordered ones[35].

**Figure 3.**
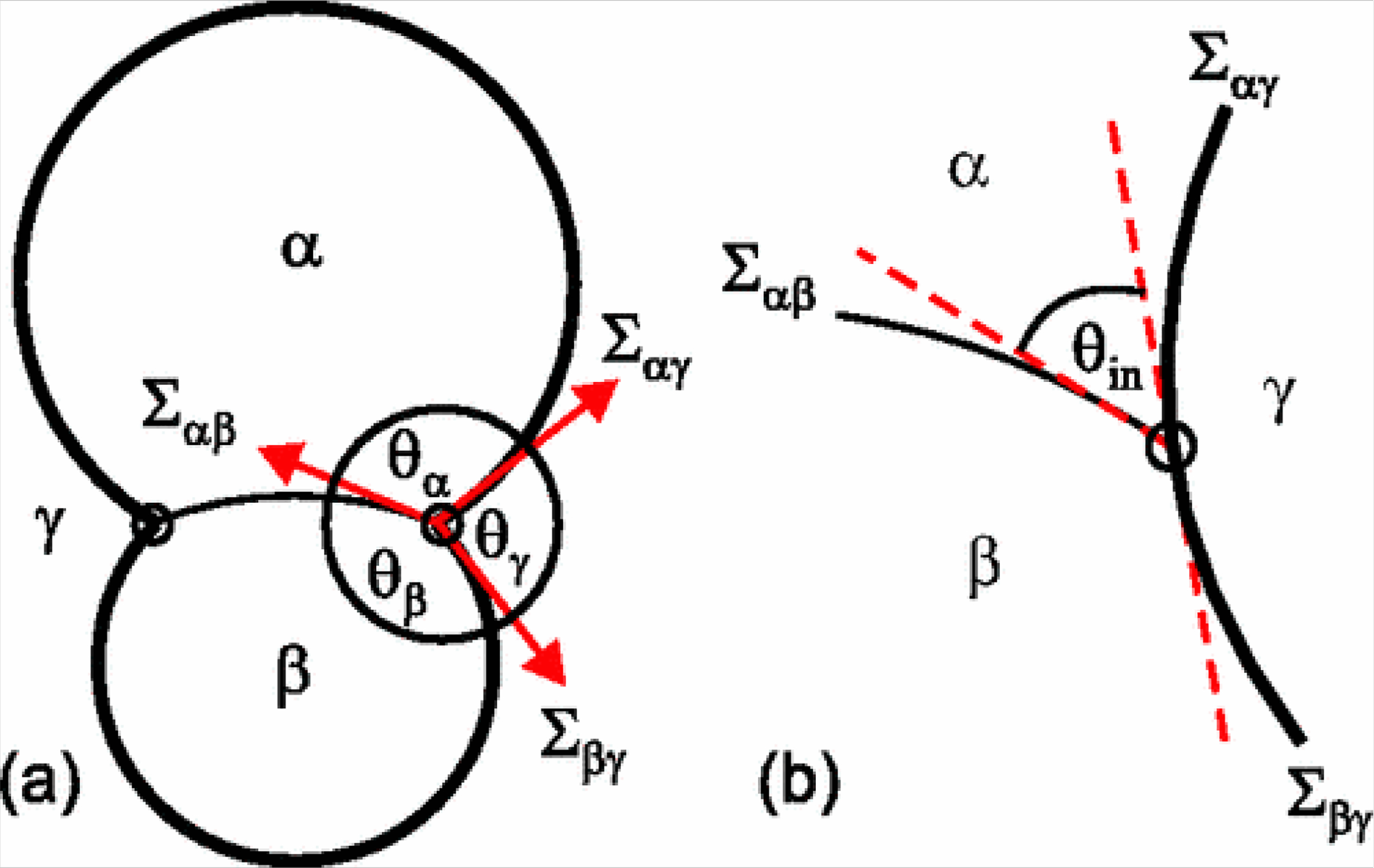
A budding vesicle encapsulating phases α and β and surrounded in phase γ. **(a)** On a macroscopic scale, three apparent contact angles (θ_α_, θ_β_, and θ_γ_) may be measured at the point where the boundary between liquid phases contacts the membrane; **(b)** The membrane is in fact locally smooth at this intersection; the intrinsic contact angle (θ_in_) is the angle between the membrane and the liquid phase boundary. Reproduced from Kusumaatmaja, Li, Dimova, and Lipowsky[36].

A parameter known as the intrinsic contact angle (θ_in_) can inform the arrangement of phases within the vesicle. At the site of budding, a “kink” appears at optical resolution to exist in the membrane which allows the measurement of three effective contact angles between the two membrane segments and the liquid-liquid phase boundary (Fig. 3a). In reality, the membrane surface must still be locally smooth at its intersection with the phase boundary, and so an intrinsic contact angle exists between the phase boundary and the membrane (Fig. 2b)[36]. This parameter, θ_in_, is a material property (unaffected by factors such as vesicle shape) if spontaneous curvature is negligible[36,37].

Nanometer-scale droplets formed through liquid-liquid phase separation can experience different physical interactions with membrane surfaces than the larger droplets discussed so far. The line tension of a droplet-surface system has an increasing impact relative to the surface tension as a droplet’s size decreases; when droplets with diameters on the scale of nanometers interact with a surface, the line tension at the contact line between droplet and surface contributes significantly to the system’s configuration[38]. In simulations, such droplets were engulfed as membrane tension is lowered, but a negative line tension broke the symmetry of the system as the membrane neck takes on a linear, lipped shape around the droplet to lengthen the contact line (Fig. 2f)[39].

### 3. Phase Separation Mediated by Membranes Can Reshape and Laterally Reorganize Membranes

#### 3.1. The Formation of Some Biomolecular Condensates is Mediated by the Membrane

Because the surface of the membrane represents a distinct environment from free solution, liquid phases may form there that do not form otherwise. Biomolecular condensates form when component concentration crosses the binodal line of the phase diagram[10], and membranes are known to locally affect protein concentrations: Proteins with no specific membrane interactions are excluded from the membrane surface and concentrated in bulk[40], and proteins may be concentrated at specific membrane sites through signaling-related protein-protein interactions[41] or curvature-sensing membrane-binding domains[42], for example. A lipid membrane has been shown to participate in the process of phase separation for a biomolecular condensate with no particular affinity for lipid membranes, possibly through confinement of condensate components or acting as a nucleation site[43]. Phase-separating proteins that can associate with (or are integral to) the membrane can form flat, lateral domains on the membrane surface. For such systems, the critical concentration for phase separation is often much lower than that for the same components in solution[44,45]. The formation of many biomolecular condensates, therefore, is mediated by the presence of a membrane, and these biomolecular condensates exhibit distinct physical interactions from droplets that phase separate from bulk solution in the absence of a membrane.

#### 3.2. Phase-Separated Protein Domains Modify the Properties of Membranes and Reshape Them

The phase separation of proteins restricted to a lipid bilayer can create spontaneous curvature by modifying the physical properties of a leaflet, leading to the reshaping of membranes. Phase separation of integral proteins and aggregation of peripheral proteins into domains on one surface of a membrane is expected to generate spontaneous curvature that can bend membranes[46]. Spontaneous curvature in membrane nanotubes has been shown to generate undulating and eventually pearling morphologies[47,48]. Simulation of a phase-separated protein domain on a nanotube demonstrated that this kind of morphological change can be driven by spontaneous curvature and by a difference in membrane stiffness introduced locally by a protein domain[49]. A recent *in vitro* study of proteins forming liquid-like domains on membranes lends support to these models: Protein domains on the exterior surface of vesicles tubulated the membrane inwards due to a compressive force that reduced the outside leaflet area, spontaneous curvature modelled the difference in leaflet area well in accompanying simulations, and the beading of the nanotubes could be controlled by modulating the stiffness of the protein domain[50]. These domains bent the vesicle membrane inward and coated the interior of the nanotube, however, unlike the aforementioned simulations of domains on the exterior of nanotubes.

This lipid bilayer leaflet compression force exhibited by a biomolecular condensate reflects the fact that membrane-mediated biomolecular condensates have the ability to laterally reorganize lipids and other bilayer components. The clearest case of this phenomenon is when a lipid is covalently bound to a phase-separating macromolecule. When cholesterol-bound polyU (chol-polyU) and poly-L-lysine were triggered to phase separate within a vesicle, the resulting biomolecular condensate remained close to the membrane, both chol-polyU and fluorescent Rh-PE lipids were concentrated in the membrane region in contact with the biomolecular condensate, and the liposome experienced a sudden and permanent decrease in size[51].

Lipid phase separation into heterogeneous membrane domains is a lateral reorganization phenomenon that is observable in giant vesicles[52–54]; it is mostly beyond the scope of this review. However, lipid and protein phase separation can affect each other: The two phenomena can promote one another, even to the point that homogenous but nearly phase-separated membranes treated with membrane-localized proteins below the phase separation concentration threshold could undergo dual phase separation, where both the lipid and protein layers phase separate[44]. One recent study proposes that the promotion of lipid phase separation by a signaling-associated membrane-bound biomolecular condensate could play a role in mediating downstream signaling[55]. Proteins in the membrane can be concentrated within (or excluded from) membrane-mediated biomolecular condensates, similar to proteins and biomolecular condensates free in solution. We will discuss this phenomenon and its functional consequences later.

#### 3.4. Membrane Reorganization by Phase-Separated Protein Domains Contributes to Biological Structures

Membrane reshaping phenomena caused by physical properties of phase-separating proteins are implicated in the formation and stability of biological membrane structures. Recent findings indicate that biomolecular condensates with viscoelastic behavior could exert force on cellular structures such as membranes[56], and one such biomolecular condensate may help form invaginations at endocytosis sites[57]. Proteins that contain BAR domains, which sense and induce membrane curvature[42], can experience a strong attraction due to the induced curvature even in the absence of specific protein-protein interactions[46]. Phase separation can arise from this kind of membrane-mediated attraction, and the phase formed from one such BAR protein likely stabilizes and amplifies membrane protrusion to produce filopodia[58]. This stabilization is partially an enhancement of the behavior of the BAR protein itself; we will next discuss more generally how biomolecular condensates modulate protein function.

### 4. Biomolecular Condensates Promoting Membrane Protein Clustering Influence Protein Function

Beyond directly, physically affecting membranes, biomolecular condensates at the surface of the membrane impact the functions of their constituent proteins and any interacting partners. This influence has the effect of mediating transmembrane signaling and subsequent membrane remodeling, as well as contributing to the formation and maintenance of long-term protein structures in the membrane. Two types of well-characterized phase-separated clusters involved in signaling are the clusters anchored by the linker for the activation of T cells (LAT) and those anchored by nephrin[3,45]. We will now discuss these and other assemblies in more detail, with a particular focus on plasma membrane signaling structures. A recent review by Zhao and Zhang catalogues the consequences of biomolecular condensate-membrane interactions from a more biological perspective, particularly interactions with endomembranes[59].

#### 4.1. Phase Separation in LAT Clusters has a Functional Contribution to T Cell Signaling

LAT is a transmembrane protein that is phosphorylated upon activation of T cell receptors (TCRs) and binds several downstream signaling elements when phosphorylated; downstream signaling does not occur in its absence[60]. LAT and some binding partners formed clusters upon TCR activation that were mediated by protein-protein interactions rather than cytoskeletal or membrane structures[61]. These clusters were phase-separated domains requiring multivalent binding partners to link LAT together. When reconstituted on a lipid bilayer, these liquid-like biomolecular condensates enriched a LAT kinase and actin polymerases and excluded a LAT phosphatase, strengthening signaling and promoting localized actin polymerization[3]. The proteins Nck and N-WASP can latch LAT domains to actin and lead to biomolecular condensate movement by different actin networks depending on the amount of Nck present, representing a model of controlling the location of biomolecular condensates and all of their components simultaneously[62].

Son of Sevenless (SOS) is one LAT binding partner necessary for phase separation whose signaling activity is activated by prolonged presence at the membrane[63]. The long average dwell time for which SOS must occupy the membrane before activation leads to a situation in which SOS is likely to be activated when part of a long-lived biomolecular condensate but unlikely to be activated when recruited to the membrane by one-off, transient interactions. This phenomenon is referred to as kinetic proofreading of LAT signaling[64]. Martin and Mittag have recently speculated that prolonged dwell times as a consequence of phase-separated signaling domains could represent a broadly applicable phenomenon, as a similar process occurs in nephrin signaling[65].

#### 4.2. Phase Separation Contributes to Nephrin Signaling

Nephrin is a transmembrane protein implicated in constructing and maintaining the slit diaphragm, an intercellular junction, and its cytoplasmic phosphorylation upon extracellular stimulation is implicated in this process[66,67]. The role of phase separation in nephrin’s signaling was first considered when its diphosphorylated tail lowered the threshold for its binding partners Nck and N-WASP to phase separate in solution, as multiple Nck proteins could assemble on the tail, effectively increasing the valency of Nck[2]. Nephrin’s phosphorylated cytoplasmic tail, when attached to a lipid bilayer, was able to form a liquid-like domain with Nck and N-WASP on the membrane, and condensed N-WASP was able to activate the actin-polymerizing Arp2/3 complex at clusters[45]. Much like SOS activation, N-WASP activation of Arp2/3 (and triggering of actin polymerization) was dependent on N-WASP dwell time at the membrane, which was not only increased greatly within clusters but was also sensitive to the nephrin/Nck/N-WASP stoichiometry[68].

#### 4.3. The Synapse is Organized by Multiple Membrane-Mediated Biomolecular Condensates

Several biomolecular condensates—including multiple membrane-associated ones—have recently been identified contributing to organization and protein function at the synapse (a recent review by Chen et al. examines these in great detail[69]). In brief, multiple distinct postsynaptic and presynaptic membrane-associated biomolecular condensates have been characterized. Several protein components of the postsynaptic density (PSD) phase-separated in solution and formed membrane clusters through association with membrane-localized NR2B (a receptor fragment). These biomolecular condensates, like those already mentioned, could promote actin polymerization, and proteins associated with inhibitory synapse PSDs were actively excluded from excitatory PSD condensates[70,71]. A set of proteins that compose inhibitory PSDs, including a scaffold and neurotransmitter receptors, have also recently been shown to phase separate on supported lipid bilayers[72]. Proteins from the presynaptic active zone have also phase-separated on the membrane by clustering calcium channels, and may keep these channels close to SNARE machinery for rapid signaling[73]. A cytoplasmic biomolecular condensate formed from synapsin was able to sequester small lipid vesicles[74], which is supported by evidence that *in vivo* synaptic vesicle clusters have liquid-like properties[75]. The sequestering of synaptic vesicles by synapsin is just one instance in which phase-separated condensates interact with small lipid vesicles; we will next discuss a variety of similar vesicle-biomolecular condensate systems.

### 5. Small Vesicles May Surround - or Act as Components of – Phase-Separated Condensates

Vesicles localize to the interior of the synapsin condensate[74] and to other biomolecular condensates as well, but small liposomes have also localized to the liquid-liquid phase boundary in some experimental conditions[76,77]. Whether vesicles localize to a biomolecular condensate surface or interior can be controlled by the intermolecular interactions that generate the biomolecular condensate and the properties of the vesicle membrane that lead to vesicle-condensate interactions.

#### 5.1. Vesicles Can Partition into a Liquid Phase of an ATPS or to the Phase Boundary

The partitioning of vesicles between two bulk aqueous phases (an ATPS) has been well-characterized for many years because it is experimentally useful for separating cell and organelle components and preparing liposomes for drug delivery use[78,79]. That research provides some background for small vesicle interactions with phase boundaries. Many studies have investigated factors that lead to liposome partitioning within one phase or another, particularly in PEG/dextran ATPSs. Such factors include pH[78,79], liposome size[80], liposome surface charge[79,80], PEGylated lipid content[81], and electric potential difference between phases[79,80]. Moldavski and Cohen in particular presented a thorough study of these and other factors in 1996[79]. These studies primarily focused on factors enhancing liposome partition to a particular phase as a method of vesicle isolation[79,82]. Liposomes have localized to the phase boundary under some conditions where they can stabilize aqueous phase droplets in a Pickering emulsion—a system in which solid particles (or in this case liposomes) stabilize colloidal liquid droplets in a liquid phase by forming a shell at the phase boundary, as opposed to the typical surfactant-stabilized emulsion[76,83,84].

#### 5.2. Vesicle Interactions with Complex Coacervates Depend on Several Physical Factors

**Figure 4.**
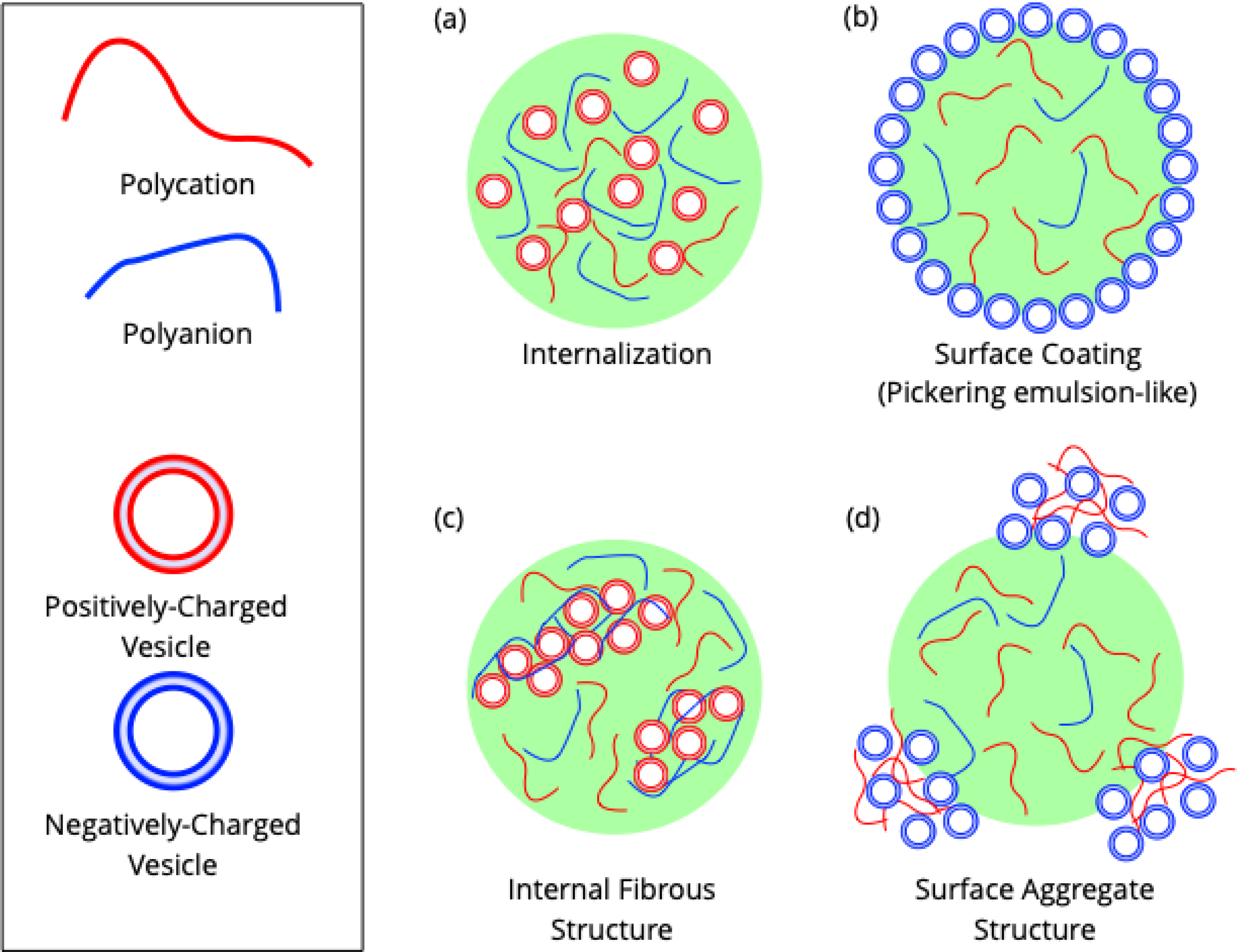
Different observed arrangements of vesicles within or around a complex coacervate. These are illustrative examples; these spatial arrangements are not definite based only on the charge of the components present. Vesicles can **(a)** be internalized within a coacervate, **(b)** coat the surface of the coacervate, **(c)** form a fibrous internal structure with a coacervate component, and **(d)** form aggregates with a coacervate component at the coacervate surface.

More recently, vesicle behavior in phase-separated systems has been investigated through interactions with droplets formed by complex coacervation. This process is an aqueous phase separation phenomenon in which a polycation and a polyanion form a dense phase driven by electrostatic interactions[85,86] as described by Overbeek-Voorn theory[17]. The simplicity and biological relevance of such droplets have made them systems of interest as models for protocells and biomolecular condensates[77,86]. The charge ratio of the coacervate (that is, the ratio of polycation to polyanion) impacts its physical properties: Coacervates with a high ratio (more polycation) have a more positive zeta potential[87,88]. Vesicles have assembled at the surface of complex coacervates, much the same as in some ATPSs (Fig. 4b). These vesicles did not fuse and did not impede exchange of RNA (a component of the coacervate) with the solution[77].

Vesicle localization in relation to complex coacervates is dependent on several factors. A recent study indicates that vesicles have a general tendency to diffuse into a complex coacervate, as their component molecules generally contain positive, negative, and hydrophobic sections[88]. That study found that negatively-charged vesicles primarily remained at coacervate surfaces with a net positive charge, but the same did not hold for positively-charged vesicles and negatively-charged coacervate surfaces (Fig. 4 a–b)[88]. This finding is consistent with previous research which used negatively-charged vesicles to generate a vesicle coating on a coacervate[77,87]. Vesicles with membranes in the gel phase rather than a liquid phase also formed a coating at the phase boundary independent of coacervate charge, presumably because they were less able to deform and so may not be able to enter the coacervate[88].

Beyond forming a uniform, one vesicle-thick layer at the phase boundary and permeation of a coacervate, vesicles have also formed aggregates with coacervate proteins at the phase boundary or inside droplets. Negatively-charged vesicles formed aggregates at the phase boundary if they could interact with polycations in the dilute phase prior to localization (Fig. 4d). Specific conditions that allowed uniformity of vesicle coating depended closely on a good “fit” between properties such as polyelectrolyte weight, charge density, charge ratio, and vesicle lipid composition[87]. At the interior of a coacervate, positively-charged vesicles could form fibrous aggregated structures with single-stranded oligonucleotides when the coacervate had a charge ratio less than or equal to one (that is, an equal or excess amount of polyanion relative to polycation) (Fig. 4c)[88].

#### 5.3. Vesicle Organization by Biomolecular Condensates is Biologically Relevant

Vesicles can be localized not only through nonspecific interactions within coacervates, but also through the presence of specific lipid-binding domains of phase-separating proteins. Synapsin binds membranes through an N-terminal region with a high affinity for anionic phospholipids[89]. RIM, a component of the presynaptic active-zone condensate, is highly positively charged, which drove negatively charged small unilamellar vesicles (SUVs) to coat the active-zone condensate surface; this may provide a mechanism for synaptic vesicle tethering[90]. A biomolecular condensate native to B cells formed by the proteins SLP65 and CIN85 also contained vesicles, which played an important role in controlling phase separation. Their presence greatly reduced the concentration threshold for biomolecular condensate formation and limited droplet size, and SLP65’s lipid-binding domain specifically bound to the highly-curved surfaces of SUVs to promote biomolecular condensate formation[91].

### 6. Active Processes and Nonequilibrium States in Cells Modify Biomolecular Condensate Properties

When biomolecular condensates are in the environment of the cell, they are subject to various means of regulation to fine-tune and control their function. Multi-droplet systems, especially in the absence of emulsifiers, are not usually in an equilibrium state. Instead, they undergo coarsening processes such as Ostwald ripening in which smaller droplets in a two-phase system diffusively lose material to larger droplets until a single large droplet remains (the true equilibrium state)[92,93]. Cells, however, maintain multi-droplet systems of condensates, leaving open the question of what mechanisms enable the maintenance in those systems of out-of-equilibrium states. One mechanism likely to be relevant in biological systems is found in active matter: Liquid-liquid phase-separated systems coupled with nonequilibrium chemical reactions (so-called “active emulsions”) can counteract Ostwald ripening and remain a monodisperse system of droplets[94]. The dynamics of the evolution of biomolecular condensate droplet systems can differ depending on a variety of different kinds of “active,” nonequilibrium chemical reactions as well as factors such as spatiotemporal differences in stimuli affecting protein affinities and undriven but reactive systems. Reviews by Berry, Brangwynne, and Haataja[95] and Lee and Wurtz[96] explore these modulations of phase separation kinetics in detail. We will turn our focus to how, specifically, membrane-associated biomolecular condensates are controlled by active processes and other related regulatory phenomena.

#### 6.1. Artificial Regulatory Mechanisms Reveal Biomolecular Condensate Responses to Nonequilibrium Processes

A simple nonequilibrium system is that of a complex coacervate droplet with a constant applied electric field. When an electric field is applied to a coacervate, a number of morphological irregularities—most notably, vacuolization (the uptake of the dilute aqueous phase)—occur[97]. Liposomes at the surface or interior of droplets under such conditions can have various effects on this behavior. Vacuolization may be suppressed, even to the point that droplet fragmentation becomes the dominant phenomenon, or it may occur and lead to the movement of surface vesicles to align with the electric field[88].

Another artificial regulatory mechanism is the fine-tuning of biomolecular condensate properties through introduction of non-native stimulus-sensitive protein domains. Membrane-bound optoDroplet proteins, which contain a light-sensitive domain that promotes phase separation when illuminated, have recently been used to demonstrate that phase separation can provide cellular “memory” of stimuli. Phase separation occurred within a locally illuminated region, but when the whole cell was subsequently illuminated, most of the droplets remained in the originally stimulated area due to diffusive principles similar to those governing Ostwald ripening[98]. A membrane-associated biomolecular condensate of endocytic proteins Eps15 and Fcho1 has recently been identified. Fine-tuning the interaction strength between these proteins through an artificial light-sensitive domain allowed researchers to generate endocytic structures that were “abortive” (highly transient), “productive,” or “stalled” (long-lived but unproductive), in order from weakest to strongest interactions[99].

#### 6.2. Regulation of Cellular Processes Impacts Biomolecular Condensates

Tuning of interaction strength is not only an artificial mechanism for regulating biomolecular condensates; it is also common biologically, in the form of post-translational modifications. A recently proposed model suggests two mechanisms of phosphorylation regulation leading to control of droplet size. In “enrichment-inhibition,” kinases that weaken interprotein interactions are enriched within biomolecular condensates, which leads to a “stable radius” for droplets where protein influx is counteracted by phosphorylative loss[100]. Those authors identified the synapsin condensate as matching these criteria[74]. In “localization-induction,” proteins are globally below the threshold for phase separation, but an immobilized kinase that enhances interprotein interactions locally increases phosphorylated protein concentrations above the threshold, generating a biomolecular condensate size-limited by the extent of kinase activity[100]. The authors identified the LAT signaling condensate as such a system[3].

Though post-translational modifications are a major method of regulation for biomolecular condensates, the cell’s control of protein synthesis and degradation also has consequences for biomolecular condensate properties, including at the membrane. A model that assumes a phase-separating molecule and a soluble counterpart that are interconverted by driven chemical reactions indicate that the formation and size of droplets can vary and Ostwald ripening even averted, depending on reaction kinetics[101]. This simple active-inactive interconversion model could be relevant to both reversible post-translational modifications and protein synthesis/degradation. A simulation of synthesis and degradation of a phase-separating particle indicates that rapid synthesis can slow the wetting of a surface like a membrane, and steady-state synthesis and degradation can also inhibit wetting[102].

## Conclusion

Here, we have reviewed a variety of interactions between phase-separated liquids and membrane surfaces, as well as the consequences of those interactions. Though the body of literature cataloging the known instances of biomolecular liquid-liquid phase separation and the various functions carried out as a consequence is vast, a focus on membrane-associated biomolecular condensates highlights a few key functions of those condensates.

Liquid-liquid phase separation has been shown to reshape membrane surfaces in a number of contexts ranging from artificial ATPSs encapsulated in vesicles to naturally phase-separating proteins coating membranes and inducing curvature. Biomolecular condensates also seem to play a role in membrane organization, both in modulating the lipid bilayer properties of regions adjacent to the condensate and in generating signaling clusters and other membrane-associated functional structures. Many of these functions are derived from biomolecular condensates’ inherent ability to sequester or exclude specific molecules. This property also allows them to sequester or otherwise organize collections of small lipid vesicles. This wide range of properties and tunability demonstrates the potential of phase-separating liquids as a highly versatile tool for life to enhance the function of the lipid membrane.
